# To Our Readers

**Published:** 1854-11

**Authors:** 


### TO OUR READERS.

We have endeavored to place the Journal into the hands of every medical man in this and of many in the surrounding States, for their examination and if they approve, we hope they will at once authorize its continuance to the P. M., who will at once give us notice if rejected. Unless this be done, we will, after a sufficient time has been allowed for the purpose, place their names permanently upon the list. We trust, for the honor and liberality of our medical brethren , the rejections will be but few if any.

During the first volume we received a notice or two that the Journal was not desired after it had been sent some time. Such a course is not just to us and certainly not honorable to those who would continue to take it for a given time, read it, and then declare they do not want it. A specimen or two of such justice is on our file of correspondence, which we shall preserve as proofs of the position that " exceptions prove the strength of a rule," and that there is " none without its exceptions."

We would respectfully call the attention of those knowing themselves in arrears for the first volume to their accounts, which will accompany this number. Our expenses are heavy and must be met promptly in order to keep the Journal in "the line of progress." The publisher must be paid and he requires his remuneration in order to meet his heavy expenditures. Every intelligent gentlemen will understand this. All we ask is to meet these requirements, for we repeat, it is no source of emolument to us. We hope that these bills will be met at once. The amount enclosed in presence of the P. M. and his certificate of the fact will satisfy us if there is any failure in reaching us. Please be particular to direct to *Iowa Medical Journal*, Box 109, *pre-paid* in all cases.

